# First application of loop‐mediated isothermal amplification (LAMP) assays for rapid identification of mating type in the heterothallic fungus *Aspergillus fumigatus*

**DOI:** 10.1111/myc.12959

**Published:** 2019-07-10

**Authors:** Kevin M. King, Nichola J. Hawkins, Sarah Atkins, Paul S. Dyer, Jonathan S. West, Bart A. Fraaije

**Affiliations:** ^1^ Rothamsted Research Biointeractions and Crop Protection Department Harpenden UK; ^2^ School of Life Sciences University of Nottingham, University Park Nottingham UK

**Keywords:** *Aspergillus fumigatus*, diagnostics, fungal pathogen, mating type, sexual reproduction

## Abstract

**Background:**

Loop‐mediated isothermal amplification (LAMP) assays, which operate at a single temperature and require no postreaction processing, have been described for rapid species‐specific detection of numerous fungi. The technology has much less commonly been applied to identification of other key genetic traits such as fungicide resistance, and has not yet been applied to mating‐type determination in any fungus.

**Objectives:**

To develop first LAMP assays for mating‐type identification in a fungus, in this instance with the saprophytic mould and human opportunistic pathogen *Aspergillus fumigatus*, a heterothallic ascomycete requiring isolates of opposite mating type (*MAT1‐1*,* MAT1‐2*) for sexual reproduction.

**Methods:**

New LAMP primer sets, targeted to *MAT* gene sequences, were screened against 34 *A fumigatus* isolates (of known mating type) from diverse clinical, environmental and geographic sources to establish whether they could distinguish *MAT1‐1* or *MAT1‐2* genotypes.

**Results and conclusions:**

The new assays, operating at a single temperature of 65°C, correctly identified the mating type of *A fumigatus* isolates in <20 minutes, and thus have numerous research and practical applications. Similar *MAT* LAMP assays could now be developed for other fungi of agricultural, environmental, industrial and/or medical importance.

## 1 INTRODUCTION

The fungus *Aspergillus fumigatus* is a saprophytic mould commonly found on plant debris and in soil. It is also an opportunistic human pathogen causing allergic symptoms and life‐threatening invasive infections. The incidence of invasive aspergillosis (IA) has been increasing in recent years largely due to increased numbers of immunocompromised individuals in the population unable to fight off infection.^1^ For more than 145 years, *A fumigatus* was only known to reproduce asexually, although several signatures of cryptic sexuality were present, for example, presence and expression of mating (*MAT*) genes and evidence of gene recombination within natural populations.^2^ However, the breakthrough 2009 discovery of a functional sexual cycle^3^ had several implications including: (a) potentially explaining high genotypic diversity observed in populations; (b) production of sexually derived airborne ascospores possibly more resilient to unfavourable environmental conditions; and (c) generation of sexual progeny with potentially greater pathogenicity and/or reduced sensitivity to fungicides.^4^ *Aspergillus fumigatus* possesses a heterothallic (obligate outbreeding) mating system, with highly dissimilar stretches of DNA, termed “idiomorphs,” present in isolates of opposite mating type as is characteristic for heterothallic ascomycete species.^5^ Thus, *MAT1‐1* isolates contain an alpha‐domain *MAT1‐1‐1* gene whereas *MAT1‐2* isolates contain a high‐mobility group *MAT1‐2‐1* gene together with a recently described *MAT1‐2‐4* gene.^6^ A multiplex PCR‐based assay for determination of mating type has previously been developed for *A fumigatus*.^2^

More recently, loop‐mediated isothermal amplification (LAMP) assays have become increasingly used for rapid species‐specific detection of numerous fungi, including *A fumigatus*.^7^ LAMP technology, first described by in 2000,^8^ typically involves 4‐6 primers in each reaction and has several purported advantages over PCR‐based diagnostics. These include faster reaction times, potentially improved sensitivity and specificity, increased tolerance of sample inhibitors, no requirement for additional postreaction processing (eg, resolving PCR products on agarose gels) and use of only a single constant reaction temperature thus raising the possibility of field‐based detection. Despite these advantages, LAMP assays have much less commonly been applied to detection of other key genetic traits such as fungicide resistance, one recent example being an assay targeted to a 34 bp tandem repeat in the *cyp51A* gene that has been associated with azole resistance in *A fumigatus*.^9^ To date, however, LAMP assays have not been used for rapid detection of different mating types in fungi. The objective of the present study was therefore to develop and evaluate for the first time whether LAMP assays could be used for the rapid identification of mating type in a fungus, with a focus here on the human opportunistic pathogen *A fumigatus*.

## 2 METHODS

### 2.1 Ethics statement

The authors confirm that the ethical policies of the journal, as noted on the journal's author guidelines page, have been adhered to. No ethical approval was required as the research in this article related to micro‐organisms.

### 2.2 Fungal isolates, DNA extraction and initial molecular characterisation

Details of *A fumigatus* isolates, including source material and geographic origin, are given in Table 2; all isolates are maintained as −80°C glycerol stocks at Rothamsted Research, UK. Genomic DNA was extracted from *A fumigatus* spores, harvested from one‐week old cultures grown on Sabouraud dextrose agar (Lab M Ltd) at 37°C, using a MasterPure yeast DNA purification kit (Epicentre) into a final volume of 100 μL TE buffer. DNA was quantified via nanodrop spectrophotometer and diluted to 10 ng/ μL using PCR‐grade water. The mating type of these isolates was first determined using the published multiplex PCR assay (Table 1).^2^ Amplicons were resolved on agarose gels, with 834 bp or 438 bp products amplified from *MAT1‐1* or *MAT1‐2* isolates, respectively.

**Table 1:** Primer sets used in the present study

Purpose/ Primer name	Primer sequence (5′ – 3′)	Source
New *MAT1‐1‐*specific LAMP assay:		
AFMAT1F3	CGGTTGGCGATATCGTGAA	Present study
AFMAT1B3	GCCATCTGTCTCTTCAGGAG	
AFMAT1FIP	CAGCGAAGGCCATTGTGGAAGTTACTGGCTACGTGTCTGAGA	
AFMAT1BIP	ACGGCATTCAGATCACTGGCGCCACTTCAGGAGTTGCGAA	
AFMAT1LOOPF	TTGGTCCGTTCGTGTGGC	
AFMAT1LOOPB	ACGATGCCATTGTGACTGAC	
New *MAT1‐2*‐specific LAMP assay:		
AFMAT2F3	CCCGTCTTGGGTAAGTGTCT	Present study
AFMAT2B3	GTGCGAAGGACTCAGTTACG	
AFMAT2FIP	CAACAGGTGCGCCAATGAGTGAGAGTTCCTCCTGAGCTTGA	
AFMAT2BIP	GCTCTCCGTGTTATGCGTACCCCAGCTTCACCGTGAGATGC	
AFMAT2LOOPF	CACTGTCATTCCGTGTTATCGG	
AFMAT2LOOPB	CAGCTTTTTCCGGAACAGCT	
Multiplex PCR mating‐type assay:		
AFM1	CCTTGACGCGATGGGGTGG	2
AFM2	CGCTCCTCATCAGAACAACTCG	
AFM3	CGGAAATCTGATGTCGCCACG	

### 2.3 Design and validation of *MAT* LAMP assays

For the *MAT1‐1* LAMP assay, *MAT* idiomorph sequence was downloaded from GenBank (Accession: AY898661 2), with LAMP primers targeted to the internal *MAT1‐1‐1* gene. For the *MAT1‐2* LAMP assay, *MAT* idiomorph sequence was sourced from the *A fumigatus* Ensembl genome (isolate AF293; gene ID: AFUA_3G06170), with LAMP primers targeted to the internal *MAT1‐2‐1* gene. LAMP primer sets (Table 1) were designed using the free online software package PrimerExplorer (v. 5) with default settings.

For screening isolates against each of the *MAT* LAMP assays, 15 μL reactions contained 0.3 μL BIP primer (final concentration 2 μmol/L), 0.3 μL FIP primer (2 μmol/L), 0.15 μL LOOPB primer (1 μmol/L), 0.15 μL LOOPF primer (1 μmol/L), 0.3 μL B3 primer (0.2 μmol/L), 0.3 μL F3 primer (0.2 μmol/L) (Table 1), μL 7.5 μL isothermal mastermix (ISO‐001; Optigene) and 1 μL DNA template (10 ng total DNA). No‐template (PCR‐grade water) controls were included in each test run. LAMP assays were run at 65°C for 30 minutes (FAM fluorescence measured every 30 seconds), followed by a final dissociation step at 95°C for 1 minutes; 55°C for 30 seconds and 95°C for 30 seconds. Assays were run with a MX3000p qPCR system (Agilent), with data analysed using inbuilt 7500 SDS software (v.1.4; Applied Biosystems). Dissociation curves were checked manually after each run to confirm the presence of a single peak.

## 3 RESULTS

### 3.1 Development and validation of *MAT* LAMP assays

**Figure 1:**
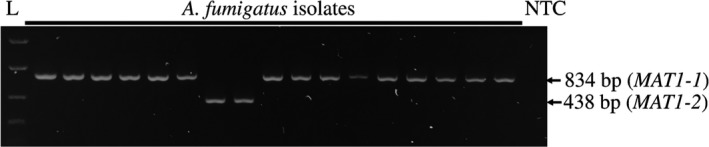
Representative results from screening of *Aspergillus fumigatus* isolates with the multiplex PCR mating‐type assay.^2^ *MAT1‐1* and *MAT1‐2* type isolates are distinguished by amplicons of 834 bp and 438 bp, respectively. “L” indicates Easyladder 1 (Bioline); NTC indicates no‐template control

**Figure 2:**
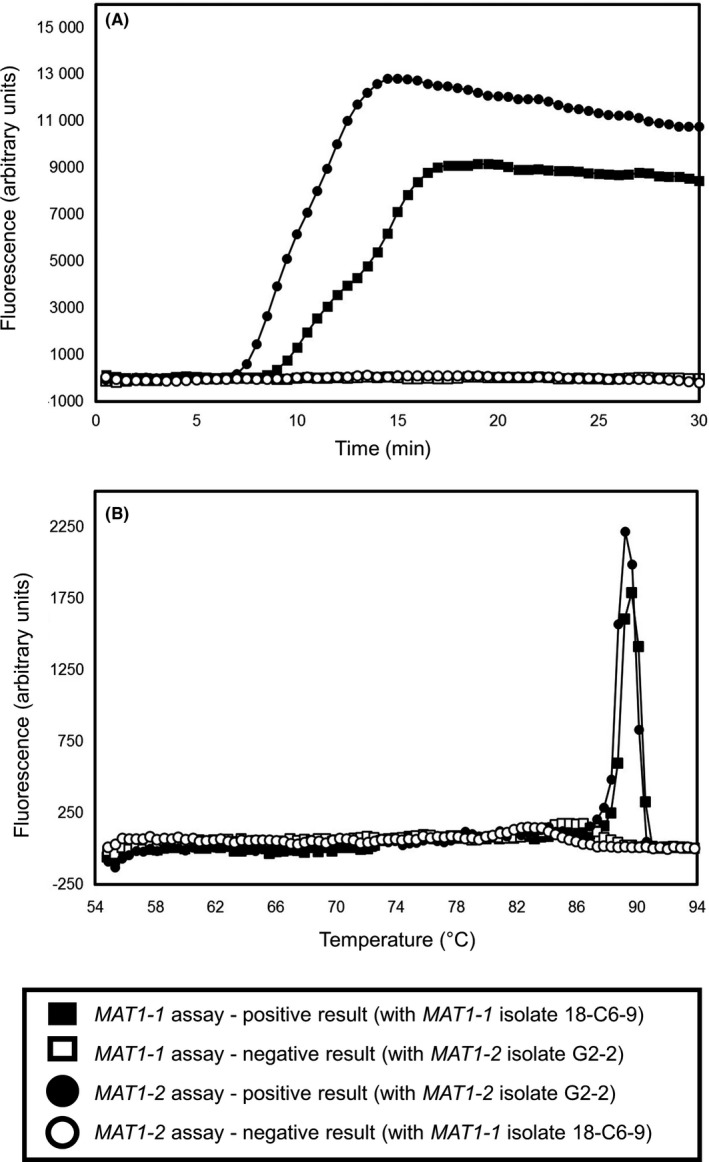
Representative results from screening of *Aspergillus fumigatus* isolates of *MAT1‐1* (18‐C6‐9) or *MAT1‐2* (G2‐2) type screened against the new *MAT*‐specific LAMP assays. Shown are (A) amplification plots and (B) dissociation plots. See base of figure for explanatory legend. No‐template (water) controls also gave negative results (data not shown)

For all *A fumigatus* isolates tested, identical *MAT* genotype results were obtained using the previously described multiplex PCR assay^2^ (see Figure 1 for representative results) and the new *MAT‐*specific LAMP assays developed in the present study (Figure 2, Table 2). The new *MAT1‐1* and *MAT1‐2*‐specific LAMP assays gave positive results within 10‐20 minutes (ie, clear amplification curves) only for isolates of corresponding *MAT1‐1* or *MAT1‐2* type, respectively (Figure 2A). Positive results obtained with each *MAT*‐specific LAMP assays gave single dissociation curves of c. 89.5°C (±0.3), indicating specific amplification of the targeted *MAT* gene regions (Figure 2B). No‐template (water) controls tested negative, that is, no amplification curves or dissociation plot peaks were observed (data not shown).

**Table 2:** Validation of new *Aspergillus fumigatus MAT* LAMP assays by screening of isolates from diverse environmental sources and geographic localities

Isolate	Source	Origin	*MAT* type (multiplex PCR)[Link]	LAMP detection time (min)	
				*MAT1‐1* assay	*MAT1‐2* assay
47‐255	Clinical	Europe	*MAT1‐1*	10‐11	Negative
47‐257	Clinical	Europe	*MAT1‐1*	9‐10	Negative
47‐258	Clinical	Europe	*MAT1‐1*	9‐10	Negative
47‐2	Clinical	North America	*MAT1‐1*	9‐10	Negative
C6‐UT1	Food	Asia	*MAT1‐1*	15‐16	Negative
C1‐2‐UT3	Food	South America	*MAT1‐1*	9‐10	Negative
C3‐UT1	Food	South America	*MAT1‐1*	9‐10	Negative
C3‐UT3	Food	South America	*MAT1‐1*	9‐10	Negative
O5‐5	Plant	Africa	*MAT1‐1*	9‐10	Negative
18‐C6‐9	Plant	Europe	*MAT1‐1*	9‐10	Negative
18‐C7‐8	Plant	Europe	*MAT1‐1*	8‐9	Negative
O9‐8	Plant	Europe	*MAT1‐1*	12‐13	Negative
T4‐1	Plant	Europe	*MAT1‐1*	15‐16	Negative
G4‐1	Plant	South America	*MAT1‐1*	8‐9	Negative
O10‐1	Plant	South America	*MAT1‐1*	19‐20	Negative
1‐2.2‐B1	Soil	Europe	*MAT1‐1*	9‐10	Negative
1‐2.2‐B2	Soil	Europe	*MAT1‐1*	9‐10	Negative
STNL1‐B1	Soil	Europe	*MAT1‐1*	9‐10	Negative
STNL1‐A8	Soil	Europe	*MAT1‐1*	9‐10	Negative
SWG1‐A9	Soil	Europe	*MAT1‐1*	12‐13	Negative
BKCb‐1	Air	Europe	*MAT1‐2*	Negative	9‐10
47‐246	Clinical	Europe	*MAT1‐2*	Negative	8‐9
Af65	Clinical	Europe	*MAT1‐2*	Negative	10‐11
Af293	Clinical	Europe	*MAT1‐2*	Negative	9‐10
C5‐T8	Food	Africa	*MAT1‐2*	Negative	9‐10
15‐37‐1	Food	Asia	*MAT1‐2*	Negative	9‐10
C1‐1‐T3	Food	South America	*MAT1‐2*	Negative	9‐10
C7‐T2	Food	South America	*MAT1‐2*	Negative	9‐10
C7‐UT1	Food	South America	*MAT1‐2*	Negative	8‐9
G2‐2	Plant	Europe	*MAT1‐2*	Negative	7‐8
SWF5‐C6	Soil	Europe	*MAT1‐2*	Negative	7‐8
PG1‐5	Soil	Europe	*MAT1‐2*	Negative	9‐10
WSN19‐3	Soil	Europe	*MAT1‐2*	Negative	9‐10
SWUK5‐A9	Soil	Europe	*MAT1‐2*	Negative	8‐9

Determined by mating multiplex PCR assay.^2^

## 4 DISCUSSION

This study reports the first use of LAMP technology to establish the mating‐type identity for a fungus, rapidly (within 20 minutes), as demonstrated here for isolates of *A fumigatus*. The *MAT1‐1* and *MAT1‐2*‐specific LAMP assays appeared robust, being successfully applied to isolates of known opposite *MAT* type from a diverse range of clinical and environmental sources (air, food, plant and soil) and geographic locations (Africa, Asia, Europe and North and South America). These assays will be of use in research into the applied biology of this important human opportunistic pathogen. For example, they will allow the rapid set‐up of sexual crosses with isolates of known opposite *MAT* type, subsequent analysis of the *MAT* type inheritance of the progeny, and through progeny analysis the determination of the genetic basis of traits such as antifungal resistance and virulence.

It should now be possible to develop similar LAMP assays targeting *MAT* gene sequences to allow rapid mating‐type determination in other heterothallic fungi of medical [eg, *Aspergillus lentulus*—another causal agent of human aspergillosis^10^], agricultural [eg, *Zymoseptoria tritici*—cause of wheat Septoria leaf blotch^11^], environmental [eg, *Hymenoscyphus fraxineus*—cause of ash dieback^12^] and industrial [eg, *Penicillium chrysogenum*—used in penicillin production^13^] importance. Such assays could also provide a better understanding into the reproductive strategies of various fungal pathogens, providing insight into their evolutionary potential and possible risk of breakdown of disease management strategies.^14^ Furthermore, they could also be used to indirectly assess possible cryptic sexuality in fungi for which no sexual stage is yet known, given that frequency dependent selection operating on *MAT* genes generally, although not always, results in a 1:1 distribution of mating types.^5^, ^14^

## CONFLICT OF INTEREST

No conflict of interest is declared.

## AUTHOR CONTRIBUTIONS

KMK, NJH, PSD, JSW and BAF conceived the ideas; KMK and SA collected the data; KMK analysed the data; KMK led the writing; all authors critically reviewed the manuscript prior to submission.
